# Alginate oligosaccharides increase boar semen quality by affecting gut microbiota and metabolites in blood and sperm

**DOI:** 10.3389/fmicb.2022.982152

**Published:** 2022-08-22

**Authors:** Hui Han, Yexun Zhou, Bohui Xiong, Ruqing Zhong, Yue Jiang, Haiqing Sun, Jiajian Tan, Bin Zhang, Chang Guan, Martine Schroyen, Liang Chen, Yong Zhao, Hongfu Zhang

**Affiliations:** ^1^State Key Laboratory of Animal Nutrition, Institute of Animal Sciences, Chinese Academy of Agricultural Sciences, Beijing, China; ^2^Department of AgroBioChem, Precision Livestock and Nutrition Laboratory, Teaching and Research Centre (TERRA), Gembloux AgroBioTech, University of Liège, Gembloux, Belgium; ^3^YangXiang Joint Stock Company, Guigang, China; ^4^Qingdao BZ Oligo Biotech Co., Ltd, Qingdao, China

**Keywords:** boar, alginate oligosaccharides, sperm motility, sperm concentration, *in vitro* fertilization, metabolism, gut microbiota

## Abstract

Alginate oligosaccharides (AOS), natural polymers from brown seaweeds (such as *Laminaria japonica*, *Undaria pinnatifida*, and *Sargassum fusiforme*), have been reported to possess many beneficial advantages for health. In the current study, after 9 weeks of dietary supplementation, AOS 10 mg/kg group (AOS 10) group increased boar sperm motility from 87.8% to 93.5%, *p* < 0.05. Moreover, AOS10 increased the relative abundances of *Bifidobacterium*, *Coprococcus*, *Butyricicoccus* (1.3–2.3-fold; *p* < 0.05) to increase the beneficial blood and sperm metabolites (1.2–1.6-fold; *p* < 0.05), and important sperm proteins such as gelsolin, Zn-alpha2 glycoprotein, Cation Channel Sperm-Associated Protein, outer dense fiber of sperm tails, etc. (1.5–2.2-fold; *p* < 0.05). AOS had a long-term beneficial advantage on boar semen quality by the increase in semen volume (175 vs. 160 ml/ejaculation, *p* < 0.05). AOS may be used as dietary additives for improving semen quality.

## Introduction

Alginate oligosaccharides (AOS), natural biodegradable polymers derived from the degradation of alginate (brown seaweed), are made up of α-L-guluronate (G) and β-D-mannuronate (M) linked by 1, 4-glycoside bonds (Hu et al., 2019). AOS have many biological advantages with the great characteristics: non-immunogenicity, non-toxicity, and biodegradability (Ueno et al., 2012; Moriya et al., 2013; Ruvinov and Cohen, 2016; Pritchard et al., 2017; Han et al., 2019; Hu et al., 2019). AOS can act as anti-inflammation (Moriya et al., 2013), anti-apoptosis (Tusi et al., 2011), anti-proliferation (Tajima et al., 1999), antioxidant activities (Tusi et al., 2011; Ueno et al., 2012; Guo et al., 2016), and even anti-cancer reagent (Yang et al., 2017). Recently, dietary AOS improved intestinal cell development and intestinal morphology and barrier function, and stimulating weaned pig growth (Wan et al., 2017, 2018a,b; Zhao et al., 2020a). Thus, AOS have been approved as a safe biopolymer by the U.S. Food and Drug Administration (reference no. 21CFR184.1724) to be applied in pharmaceutical, cosmeceutical, and nutraceutical fields (Park et al., 2016; Hu et al., 2019).

Reproductive biotechnologies, especially artificial insemination (AI), play a vital role in the genetic improvement of pigs and other farm animals (Singh et al., 2021). Moreover, AI not only makes significant contributions to the development of swine production worldwide, it also raises the importance of the reproductive efficiency of boars in pig herds (Tsakmakidis et al., 2010). Semen quality is used as a proxy measure of boar fertility owing to the close correlation between sperm quality with boar fertility, and it creates a desired effect on piglet production in terms of the reproductive performance of sows (Dong et al., 2016). In summary, good semen quality is fundamental for successful AI (Tsakmakidis et al., 2010; Singh et al., 2021).

The production and quality of semen not only depend on intrinsic factors such as breed (Wolf, 2009) and age (Huang et al., 2010), but also on environmental extrinsic factors, for example, temperature, photoperiod, and nutrition (Ciereszko et al., 2000; Yeste et al., 2010; Dong et al., 2016). Boar age and semen quality are factors that are considered in boar culling (Tsakmakidis et al., 2012). Reports show that a maximum semen volume and sperm concentration can be obtained from boars of ≤3.5 years of age (Smital, 2009; Tsakmakidis et al., 2012). Hormonal and cellular changes take place in males with aging, which alters sperm quality and fertilization capacity (Araujo and Wittert, 2011). Aging also induces a decrease in testosterone levels, which is involved with intrinsic and extrinsic factors associated with Leydig cells (Midzak et al., 2009; Araujo and Wittert, 2011; Tsakmakidis et al., 2012).

Nutrition influences boar libido, sperm output, semen quality, and fertility (sow pregnancy rate and litter sizes; Dong et al., 2016; Liu et al., 2017a). It is known that protein levels in the diet affect boar semen quality; both low protein or excessive protein can decrease sperm quality (Louis et al., 1994; Dong et al., 2016). Individual amino acids have potential impacts on semen quality (Ren et al., 2015) as follows: dietary lysine (1.03%) improves boar semen quality compared to 0.86% (Dong et al., 2016); supplementation of threonine benefits ram and boar sperm quality (Wilson et al., 2004); tryptophan significantly improves ram sperm motility (Pichardo et al., 2011; Dong et al., 2016). Furthermore, polyunsaturated fatty acids (PUFAs) have been shown to benefit sperm motility and fertility in human and animal studies (Murphy et al., 2017; Liu et al., 2017a). The major commercial source of omega-3 fatty acids [specifically docosahexaenoic acid (DHA)] are fish oils, and the most abundant source for linolenic acid is flaxseed (~53%). Chestnut polysaccharides have been discovered to improve the spermatogenesis and semen quality recently (Yu et al., 2020; Sun et al., 2022). With the great advantages, the purpose of this investigation was to explore AOS benefit boar semen quality and the underlying mechanisms.

## Materials and methods

### Materials and reagents

AOS (>98%) was from Qingdao BZ Oligo Biotech Co. Eosin, insulin, EGF, cysteine, pyruvate, kanamycin, paraformaldehyde, and Triton X-100 were from Sigma-Aldrich. BSA, goat serum, and TCM-199 medium were from Life Technologies Ltd. Polyvinylidene fluoride (PVDF) membrane was from Merck. E.Z.N.A.^®^ Stool DNA Kit was from Omega Bio-tek Inc. The antibodies were purchased from different companies listed in Supplementary Table S1.

### Boars and experimental design

**Figure 1:**
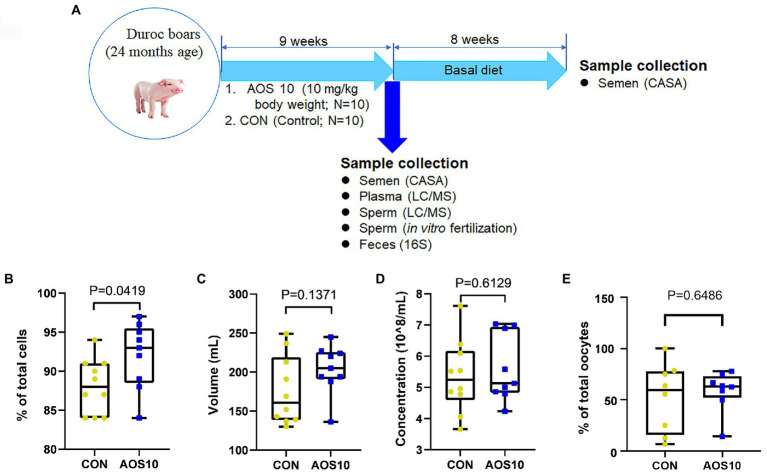
The impacts of AOS on boar sperm quality. **(A)** Study scheme. **(B)** Spermatozoa motility determined by CASA. Y-axis = % of total cells, X-axis = treatment group (mg/kg body weight). *n* = 10. **(C)** Semen volume. Y-axis = volume (ml), X-axis = treatment concentration (mg/kg body weight). *n* = 10. **(D)** Sperm concentration. Y-axis = sperm concentration (10^8/ml), X-axis = treatment group (mg/kg body weight). *n* = 10. **(E)** *In vitro* fertilization rate. Y-axis = % of total oocytes, X-axis = treatment group (mg/kg body weight). *n* = 10.

All animal procedures were approved by the Animal Care and Use Committee of the Institute of Animal Sciences of Chinese Academy of Agricultural Sciences (IAS2021-67). Twenty boars (~24 months of age; male) were used in this investigation at the artificial insemination center of Yangxiang Joint Stock Company (Guangxi, China; Guo et al., 2020). Boar feeding conditions have been previously reported (Supplementary Table S2; Wu et al., 2019a). Our preliminary study has found that 10 mg/kg was good concentration for improving boar semen quality. There were two treatments: (1) Control group (CON), 10 boars fed with a basal diet, and (2) AOS 10 mg/kg group (AOS 10), 10 boars fed with a basal diet plus 10 mg/kg body weight of AOS. Semen samples were collected after 9-week feeding (Figure 1A) by gloved-hand techniques. After collection, four semen parameters were assessed: semen volume, sperm concentration, sperm motility, and abnormal sperm rate, according to the reported methods (Wu et al., 2019b; Guo et al., 2020). Blood samples were harvested by venipuncture from the hindlimb vein of boars during ejaculations. Each blood sample was then centrifuged at 3000× g for 10 min at 4°C to obtain a plasma sample and subsequently stored at −80°C until analysis. Each boar’s rectum was massaged to stimulate defecation, and then, fresh feces were collected and stored at −80°C for subsequent microbiota analysis (Guo et al., 2020). The long-term effects of AOS on boar semen quality were determined. After AOS supplementation, all the boars were fed with basal diet (without AOS supplementation) for another 8 weeks. The semen was collected every 5 days and the semen quality was analyzed (Figure 1A).

### Evaluation of spermatozoa motility using a computer-assisted sperm analysis system

Spermatozoa motility and concentration were determined by the computer-assisted sperm assay (CASA) method according to World Health Organization guidelines (WHO, 2010; Zhao et al., 2016; Zhang et al., 2018, 2019). Boar spermatozoa were incubated at 37.5°C for 30 min then samples were placed in a pre-warmed counting chamber (MICROPTIC S.L., Barcelona, Spain). The Microptic Sperm class analyzer (CASA system) was used in this investigation. It was equipped with a 20-fold objective, a camera adaptor (Eclipse E200, Nikon, Japan), and a camera (acA780-75gc, Basler, Germany); it was operated by an SCA sperm class analyzer (MICROPTIC S.L.).

### Morphological observations of spermatozoa

Boar sperm was stained with Eosin Y (1%; Zhao et al., 2016; Zhang et al., 2018, 2019). Spermatozoa abnormalities were then viewed using a bright-field microscopy (AH3-RFCA, Olympus, Tokyo, Japan) and were classified into head or tail morphological abnormalities: two heads, two tails, blunt hooks, and short tails. The examinations were repeated three times, and 500 spermatozoa per animal were scored.

### *In vitro* fertilization

The procedure for the preparation of porcine oocytes has been reported previously (Redel et al., 2019; Zhou et al., 2020). Porcine ovaries were obtained from a slaughterhouse. Follicular fluid from 3 to 6 mm antral follicles was aspirated with an 18-gauge syringe. Cumulus oocyte complexes (COCs) with uniform cytoplasm and several layers of cumulus cells were selected and rinsed three times in washing medium [TCM-199 medium supplemented with 10% porcine follicular fluid (pFF), 5 μg/ml insulin, 10 ng/ml EGF, 0.6 mM cysteine, 0.2 mM pyruvate, and 25 μg/ml kanamycin]. Approximately 70 COCs per well were cultured under mineral oil in 4-well plates containing TCM-199 medium supplemented with 10% porcine follicular fluid (pFF), 5 μg/ml insulin, 10 ng/ml EGF, 0.6 mM cysteine, 0.2 mM pyruvate, 25 μg/ml kanamycin, and 5 IU/ml of each of eCG and hCG. The oocytes were matured for 44 h at 38.5°C, 5% CO_2_ in a humidified incubator.

The *in vitro* fertilization (IVF) medium Tyrode’s albumin lactate pyruvate (TALP) 29 was previously equilibrated for ~3 h at 38.5°C, with 5% CO_2_ in air and a humidified incubator until it reached a final pH of 7.4. *In vitro* mature (IVM) oocytes were mechanically denuded with an automatic pipette, washed in TALP medium, and transferred in groups of 50 oocytes to a 4-well plate (Nunc, Roskilde, Denmark) containing 500 μl TALP medium per well. Sperm suspensions were added to the IVF wells at a final concentration of 25 ◊ 10^3^ cells/ml. After a 6-h coculture, the putative zygotes were fixed with 0.5% glutaraldehyde in phosphate-buffered saline (PBS), stained with 1% (w/v) Hoechst 33342 in PBS, and examined under an epifluorescence microscope. The parameters analyzed were the percentage of oocytes penetrated by one or more spermatozoa (Pen, %; Zapata-Carmona et al., 2020).

### Boar fecal microbiota analysis

#### DNA extraction

Total genomic DNA of boar feces was isolated using an E.Z.N.A.^®^ Stool DNA Kit (Omega Bio-tek Inc., United States) following the manufacturer’s instructions. DNA quantity and quality were analyzed using NanoDrop 2000 (Thermo Scientific, United States) and 1% agarose gel (Zhang et al., 2020).

#### Library preparation and sequencing

The V3–V4 region of the 16S rRNA gene was amplified using the primers 338F (5′-ACTCCTACGGGAGGCAGCAG-3′) and 806R (5′-GGACTACHVGGGTWTCTAAT-3′) with Barcode. The PCR reactions (total 30 μl) included 15 μl Phusion^®^ High-Fidelity PCR Master Mix (New England Biolabs), 0.2 mM primers, and 10 ng DNA. The thermal cycle was carried out with an initial denaturation at 98°C, followed by 30 cycles of 98°C for 10 s, 50°C for 30 s, 72°C for 30 s, and a final extension at 72°C for 5 min. PCR products were purified using an AxyPrep DNA Gel Extraction Kit (Axygen Biosciences, United States). The sequencing libraries were constructed with NEB Next^®^ Ultra^TM^ DNA Library Prep Kit for Illumina (NEB, United States) following the manufacturer’s instructions, and index codes were added. Then, the library was sequenced on the Illumina MiSeq 2,500 platform (Illumina, United States) and 300 bp paired-end reads were generated at the Novo gene. The paired-end reads were merged using FLASH (V1.2.71; Magoč and Salzberg, 2011). The quality of the tags was controlled in QIIME (V1.7.02); meanwhile, all chimeras were removed (Zhang et al., 2020). The “Core Set” of the Greengenes database3 (DeSantis et al., 2006) was used for classification, and sequences with >97% similarity were assigned to the same operational taxonomic units (OTUs).

#### Analysis of sequencing data

OTU abundance information was normalized using a standard of sequence number corresponding to the sample with the least sequences. The alpha diversity indices were calculated with QIIME (Version 1.7.0; Caporaso et al., 2010). Partial least squares discrimination analysis (PLS-DA) was performed using R software (v2.15.3).

### Plasma and sperm metabolites determination

Plasma and sperm metabolites were determined by LC–MS/MS (Zhang et al., 2020). Boar plasma and sperm were collected and maintained at −80°C. The protein was removed from the samples before LC–MS/MS analysis with ACQUITY UPLC and AB Sciex Triple TOF 5600 (LC/MS) as reported previously (Zhang et al., 2020).

The conditions for HPLC were: ACQUITY UPLC BEH C18 column (100 mm × 2.1 mm, 1.7 μm), solvent A [aqueous solution with 0.1% (v/v) formic acid], and solvent B [acetonitrile with 0.1% (v/v) formic acid] with a gradient program: 0–2 min, 5%–20% B; 2–4 min, 20%–25% B; 4–9 min, 25%–60% B; 9–17 min, 60%–100% B; 17–19 min, 100% B; 19–19.1 min, 100%–5% B; and 19.1–20.1 min, 5% B. The flow rate was set at 0.4 ml/min and 5 μl was injected. ESI was used in the mass spectrometry program. Progenesis QI v.2.3 (Nonlinear Dynamics, Newcastle, United Kingdom) was used to normalize the peaks. Human Metabolome Database (HMDB), LIPID MAPS (v. 2.3), and METLIN software were used to qualify the data. Furthermore, the data were analyzed with SIMCA software (v. 14.0, Umetrics, Umeå, Sweden) and the KEGG database^1^ was used for pathway enrichment analysis.

### Detection of protein levels and location in spermatozoa using immunofluorescence staining

The methods for IHF of boar sperm have been reported in our previous articles. Boar spermatozoa were fixed in 4% paraformaldehyde for 1 h, then the cells were spread onto poly-L-lysine coated microscope slides and air-dried. After three washings with PBS (5 min each), spermatozoa were incubated with 2% (vol/vol) Triton X-100 in PBS for 1 h at RT. Then, after three washes with PBS, the cells were blocked with 1% (wt/vol) BSA and 1% goat serum in PBS for 30 min at RT, followed by incubation with primary antibodies (1:100; Supplementary Table S2) diluted in blocking solution overnight at 4°C. The following morning, after three washes with PBS Tween 20 (0.5%) the slides were incubated with Alexa Fluor 546 goat anti-rabbit IgG (1,200) for 30 min in darkness at RT. The negative control samples were incubated with a secondary antibody and without a primary antibody. Slides were washed with PBS Tween-20 three times and then incubated with DAPI (4.6-diamidino-2-phenylindole hydrochloride, 100 ng/ml) as a nuclear stain for 5 min. After a brief wash with ddH_2_O, the slides were covered with an anti-fading mounting medium (Vector, Burlingame, United States). Fluorescence images were obtained with a Leica Laser Scanning Confocal Microscope (LEICA TCS SP5 II, Germany; Zhao et al., 2016; Zhang et al., 2020).

### Determination of protein levels by western blotting

The procedure for Western blotting analysis of boar sperm proteins is reported in our previous publications (Zhao et al., 2016; Zhang et al., 2020). Briefly, sperm cells were lysed in RIPA buffer containing the protease inhibitor cocktail from Sangon Biotech, Ltd. (Shanghai, China). Protein concentration was determined using a BCA kit (Beyotime Institute of Biotechnology, Shanghai, China). Actin was used as a loading control. The primary antibodies (Abs) are listed in Supplementary Table S2. Secondary donkey anti-goat Ab (Cat no.: A0181) was purchased from Beyotime Institute of Biotechnology (Shanghai, P.R. China), and goat anti-rabbit (Cat no.: A24531) Abs were bought from Novex^®^ by Life Technologies (United States). Fifty micrograms of total protein per sample were loaded onto 10% SDS polyacrylamide electrophoresis gels. The gels were transferred to a polyvinylidene fluoride (PVDF) membrane at 300 mA for 2.5 h at 4°C. Then, the membranes were blocked with 5% BSA for 1 h at RT, followed by three washes with 0.1% Tween-20 in TBS (TBST). The membranes were incubated with primary Abs diluted at 1:500 in TBST with 1% BSA overnight at 4°C. After three washes with TBST, the blots were incubated with the HRP-labeled secondary goat anti-rabbit or donkey anti-goat Abs, respectively, for 1 h at RT. After three washes, the blots were imaged.

### Statistical analysis

All data were expressed as mean ± standard deviation (SD). Data were determined by SPSS statistical software (IBM Co., NY) with one-way analysis of variance (ANOVA), followed by LSD multiple comparison test. Graphs were created using GraphPad Prism 5.20 (GraphPad Software Inc., La Jolla, CA, United States). All groups were compared with each other for every parameter. *p* < 0.05 was considered to be significant.

### Data availability

The microbiota raw sequencing data generated in this study has been uploaded to the NCBI SRA database with the accession number GSE178723.

## Results

### Impact of AOS on boar semen quality and *in vitro* fertility potential

After 9 weeks of feeding (Figure 1A), the AOS10 group showed significantly increased sperm motility from 87.8% to 93.5% (Figure 1B; *p* < 0.05). The semen volume per boar per day (204 ml/ejaculation vs. 176 ml/ejaculation; *p* = 0.612) and the concentration (5.6 × 10^8/ml vs. 5.0 × 10^8/ml; *p* = 0.137) of sperm also showed an increasing trend in AOS10 over CON while the difference was not significant (Figures 1C,D). Moreover, the *in vitro* fertilization rate was also higher in AOS10 (58.6%) than in CON (52.1%; Figure 1E) while it was not significant.

### Effects of AOS on boar sperm quality and sperm metabolism

**Figure 2:**
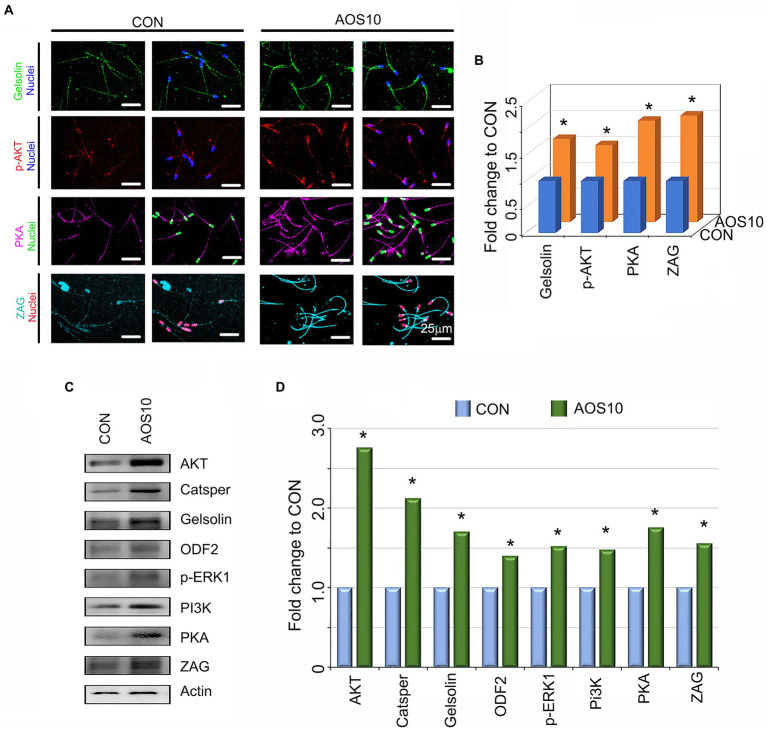
The influence of AOS on the protein expression in boar sperm. **(A)** Sperm protein levels of important genes for semen quality as detected by immunofluorescence staining. *N* > 6. **(B)** Quantitative data for immunofluorescence staining. Y-axis = fold change to CON, X-axis = treatment group (mg/kg body weight). ^*^p < 0.05. **(C)** Sperm protein levels of important genes for semen quality as detected by Western blotting. *N* > 3. **(D)** Quantitative data for western blotting analysis. Y-axis = fold change to CON, X-axis = treatment group (mg/kg body weight). ^*^p < 0.05.

To understand the mechanisms underlying the AOS improvement of semen quality, we explored the protein levels of important genes for sperm quality. AOS10 increased the protein levels of gelsolin (1.61-fold), p-AKT (phosphorylated protein kinase B; 1.48-fold), protein kinase A (PKA; 1.95-fold), and Zn-alpha2 glycoprotein (ZAG; 2.05-fold) according to IHF detection (Figures 2A,B; ^*^p < 0.05). The data for these protein levels were confirmed by western blotting analysis. Moreover, the levels of the other four sperm proteins such as cation channel sperm-associated protein (CatSper; 2.12-fold), outer dense fiber of sperm tails 2 (ODF2; 1.42-fold), p-ERK1 (phosphorylated extracellular signal-regulated kinase; 1.51-fold 1), and Phosphoinositide-3-Kinase (PI3K; 1.47-fold) were also elevated in AOS10 over CON (Figures 2C,D; ^*^p < 0.05).

**Figure 3:**
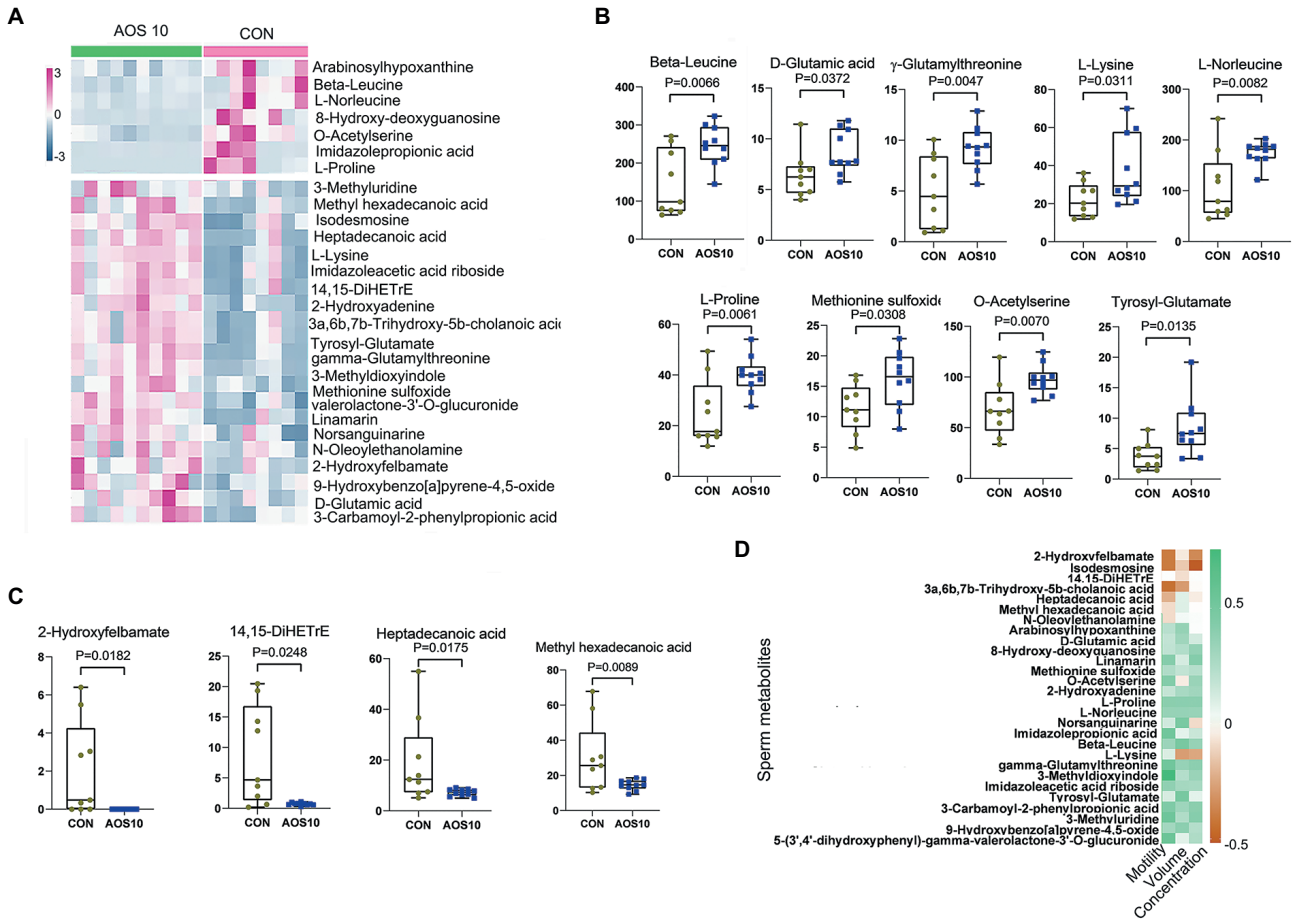
The impacts of AOS on boar sperm metabolome. **(A)** Heatmap of changed sperm metabolites. The sperm metabolites were determined by LC–MS/MS. **(B)** List of AOS increased sperm metabolites. Y-axis = relative amount, X-axis = treatment group (mg/kg body weight). **(C)** List of AOS decreased sperm metabolites. Y-axis = relative amount, X-axis = treatment group (mg/kg body weight). **(D)** Correlation of sperm metabolites and sperm concentration, volume, and motility.

There were 1,031 metabolites detected in the boar sperm samples (Supplementary Figure S1; Supplementary Table S3). The OPLS-DA analysis showed that the AOS10 and CON groups were well separated (Supplementary Figure S1A). Twenty-eight metabolites were significantly different in AOS10 compared to CON (Figure 3A; Supplementary Tables S4, S5). Nine of the significantly increased metabolites including β-leucine, D-glutamic acid, γ-glutamylthreonine, L-lysine, L-norleucine, L-proline, methionine sulfoxide, O-acetylserine, and tyrosyl-glutamate are shown in Figure 3B (1.2–1.6folds; *p* < 0.05). Some of the significantly decreased metabolites (such as 2-hydroxylfelbamate, 14,15-diHETrE, heptadecanoic acid, and methyl hexadecenoic acid) are shown in Figure 3C (5%–10%; *p* < 0.05). These 28 metabolites were well correlated with sperm motility, sperm concentration, and sperm volume (Figure 3D), with the increased metabolites in AOS10 being positively correlated with these sperm parameters, while the decreased metabolites in AOS10 were negatively correlated. At the same time, the metabolites were well correlated with each other (Supplementary Figure S1D).

### Impact of AOS on boar blood metabolism

**Figure 4:**
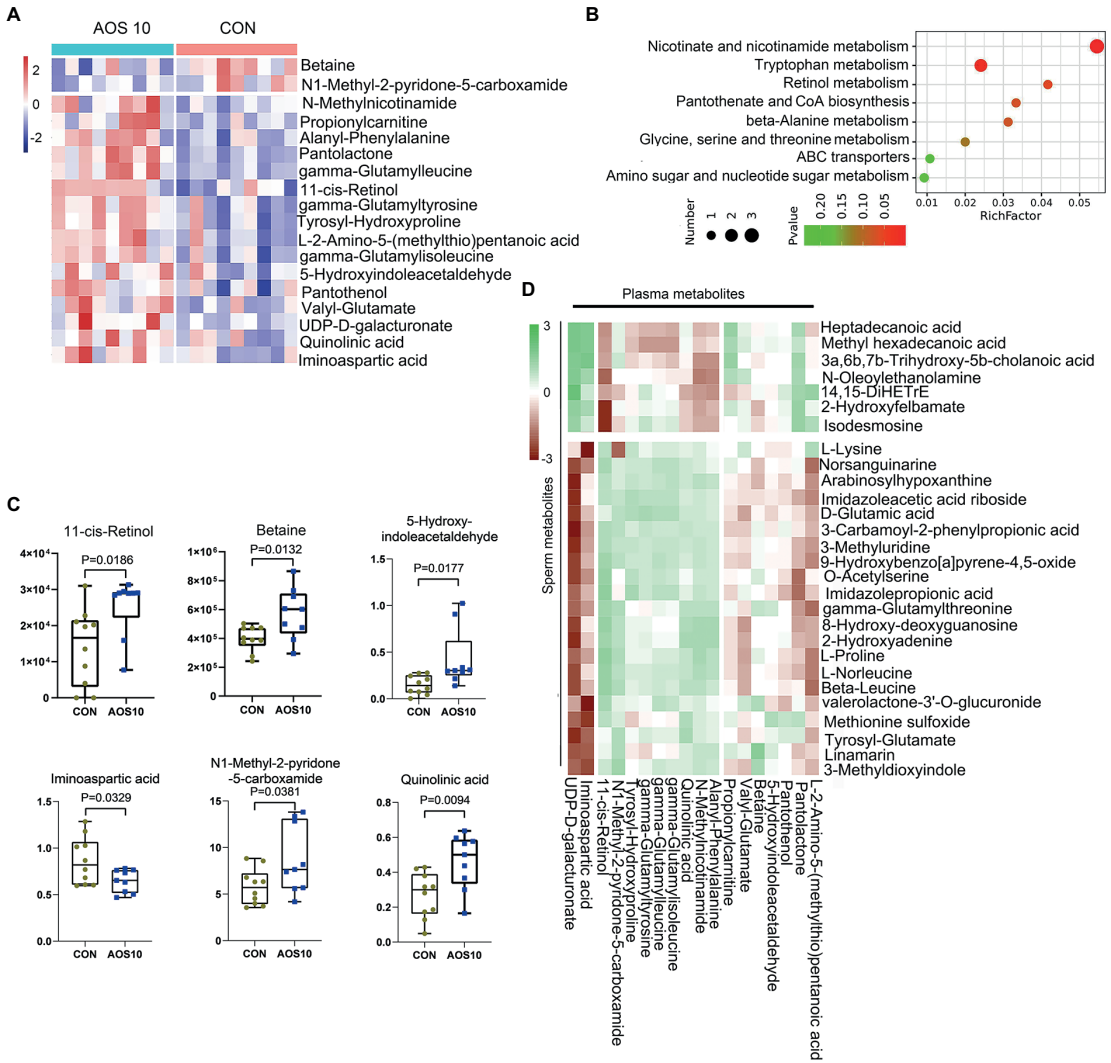
The effects of AOS on boar blood metabolome. **(A)** Heatmap of changed blood metabolites. The blood metabolites were determined by LC–MS/MS. **(B)** KEGG enriched pathways of changed blood metabolites. **(C)** List of AOS changed blood metabolites. Y-axis = relative abundance, X-axis = treatment group (mg/kg body weight). **(D)** Correlation of blood metabolites and sperm metabolites.

There were 1,087 metabolites detected in boar plasma samples (Supplementary Figure S2; Supplementary Table S6). The OPLS-DA analysis showed that the AOS10 and CON groups were well separated (Supplementary Figure S2A). Eighteen metabolites were significantly different in AOS10 compared with CON (Figure 4A; Supplementary Tables S7, S8). Functional enrichment of these metabolites showed that they were involved in amino acid metabolism and retinol metabolism (Figure 4B). Some of the significantly changed metabolites are shown in Figure 4C. 11-cis-retinol, betaine, 5-hydroxy-indoleacetaldehyde, N1-methyl-2-pyridone-5-carboxamide, and quinolinic acid were increased from 1.5–2.4-fold (*p* < 0.05), while iminoaspartic acid was decreased (45%, *p* < 0.05). At the same time, these metabolites were well correlated with each other (Supplementary Figure S2C). Moreover, the significantly changed blood metabolites and the significantly changed sperm metabolites were well correlated (Figure 4D; Supplementary Table S9).

### Effects of AOS on boar gut microbiota

**Figure 5:**
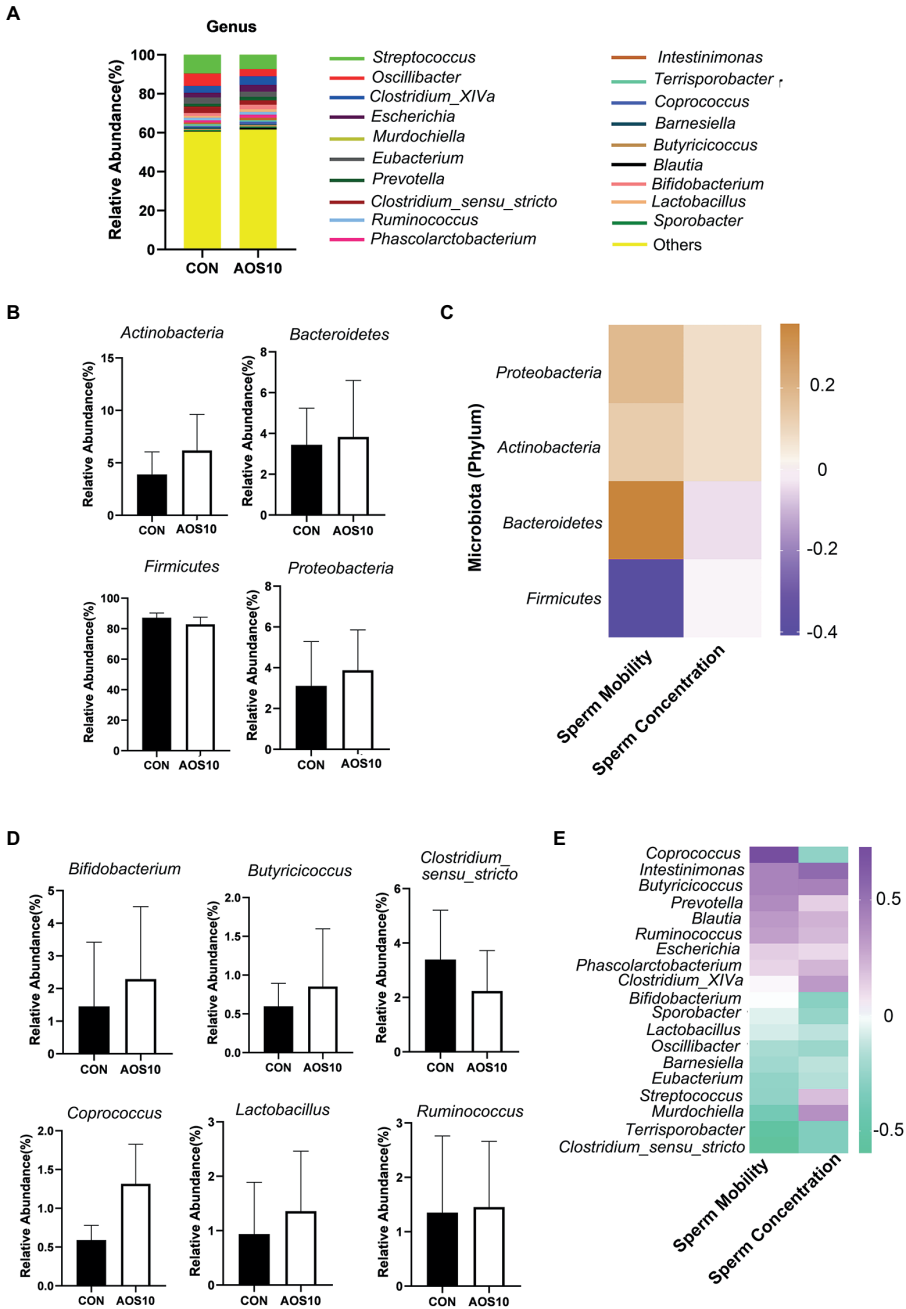
The influence of AOS on boar gut microbiota. **(A)** Differences of bacterial abundance at the genus level. Y-axis = relative abundance, X-axis = treatment group (mg/kg body weight). **(B)** Differences of bacterial abundance at the class level. **(C)** Correlation of bacterial abundance at the class level with sperm motility and concentration. **(D)** Representative differences of bacterial abundance at the genus level. Y-axis = relative abundance, X-axis = treatment group (mg/kg body weight). **(E)** Correlation of bacterial abundance at the genus level with sperm motility and concentration.

AOS10 affected the gut microbiota composition (Figure 5; Supplementary Figure S3). At the phylum level, compared to CON, AOS10 increased the relative abundances of *Bacteroidetes* (1.21fold; *p* > 0.05), decreased the levels of *Firmicutes* (Figure 5B; *p* > 0.05), and increased the ratio of *Bacteroidetes/Firmicutes* (Supplementary Figure S3E) in boar fecal samples. Moreover, at the class level, the microbiota and sperm motility were well correlated and *Bacteroidetes* was positively correlated with sperm motility; meanwhile, *Firmicutes* was negatively correlated with sperm motility (Figure 5C). At the genus level, AOS10 increased the relative abundances of several beneficial bacteria: *Bifidobacterium* (1.51-fold), *Coprococcus* (2.33-fold), and *Butyricicoccus* (1.33-fold). (Figure 5D; *p* > 0.05). Moreover, *Coprococcus* was positively correlated with sperm motility (Figure 5E), while *Intestinimonas* and *Lactobacillus* were positively correlated with both sperm motility and concentration (Figure 5E).

**Figure 6:**
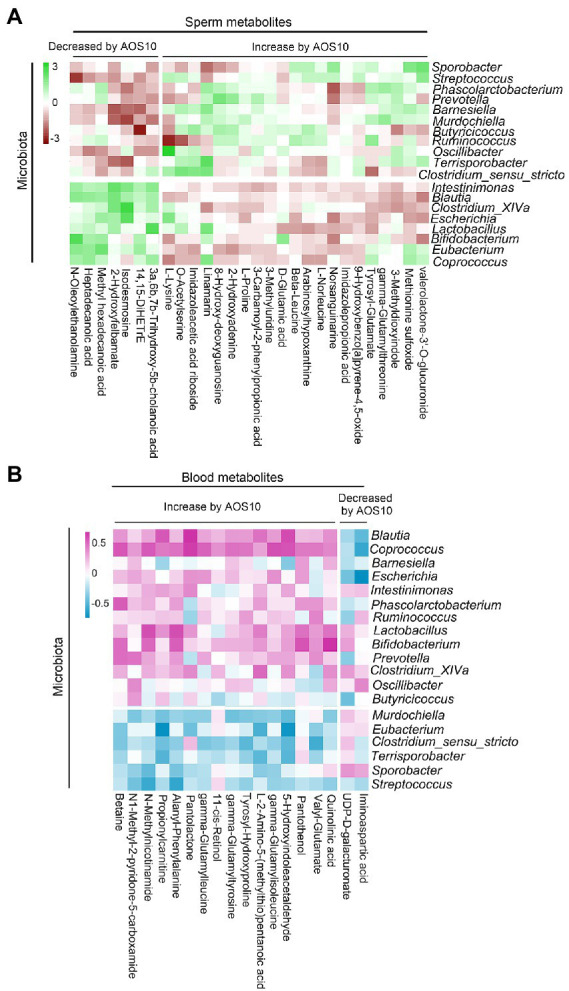
**(A)** Correlation of sperm metabolites with fecal microbiota. **(B)** Correlation of blood metabolites with fecal microbiota.

Furthermore, sperm metabolites and fecal microbiota were well correlated (Figure 6A; Supplementary Table S10). Sperm metabolites increased in the AOS10 group were positively correlated with the beneficial microbiota *Bifidobacterium*, *Coprococcus*, and *Butyricicoccus* (Figure 6A). Similarly, blood metabolites and fecal microbiota were well correlated (Figure 6B; Supplementary Table S11), and the blood metabolites increased in AOS10 were positively correlated with the beneficial microbiota *Bifidobacterium*, *Coprococcus*, and *Lactobacillus* (Figure 6B).

### Impact of AOS10 on semen quality for a long time

**Figure 7:**
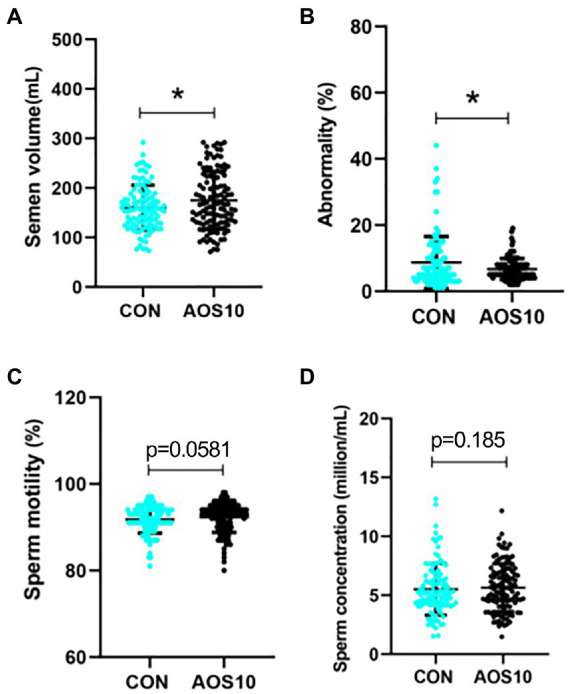
Long-term effects of AOS on boar semen quality. After AOS supplementation, boars were fed with basal diet for 8 weeks. Semen quality was determined every 5 days for 2 months (no AOS supplementation). **(A)** Semen volume. Y-axis = volume (ml), X-axis = treatment group (mg/kg body weight). **(B)** Abnormal sperm. Y-axis = % of total cells, X-axis = treatment group (mg/kg body weight). **(C)** Sperm motility. Y-axis = % of total cells, X-axis = treatment group (mg/kg body weight). **(D)** Sperm concentration. Y-axis = sperm concentration (10^8/ml), X-axis = treatment group (mg/kg body weight). *n* = 10, ^*^p < 0.05.

AOS had a long-term beneficial improvement on boar semen quality by the increase in the semen volume (175 vs. 160 ml/ ejaculation, *p* < 0.05) and sperm motility, while the decrease in the percentage of abnormal sperm after another 2 months on basal diet (without AOS addition; Figures 7A–C). The sperm concentration was in an increased trend in AOS10 group compared to CON (Figure 7D; *p* = 0.185).

## Discussion

AOS have been used in many different perspectives as anti-inflammation (Moriya et al., 2013), anti-apoptosis (Tusi et al., 2011), anti-proliferation (Tajima et al., 1999), and even anti-cancer chemicals (Wan et al., 2017) because of the highly desired natural properties (non-immuno-genicity, biodegradability, and non-toxicity). AOS decrease the production of nitric oxide, prostaglandin E2, and pro-inflammatory cytokines, and block the expression of toll-like receptor 4 and nuclear factor (NF)-κB to prevent neuroinflammation or neurodegenerative diseases (such as Alzheimer’s disease; Zhou et al., 2015). Wan and coauthors reported that AOS improved antioxidant levels and increased piglet growth rates (Wan et al., 2017, 2018a,b). AOS benefited intestinal epithelial cell growth and differentiation to improve livestock growth rates (Zhao et al., 2020a). Moreover, AOS improved busulfan disrupted spermatogenesis in mice (Zhao et al., 2020b). In the current investigation, we found that AOS 10 mg/kg could benefit boar sperm motility. The difference between AOS10 and CON was small, which may be because the boars used in this investigation were at an optimal age for sperm production; therefore, the room for improvement in these parameters was limited. However, altogether, the data suggested that AOS has the potential to improve boar semen quality and fertility. Moreover, AOS increased the levels of important proteins in boar sperm such as gelsolin, ODF2, PKA, AKT, etc. All these proteins play important roles in sperm function or fertility (Lee, 2012; Liu et al., 2012; Finkelstein et al., 2013; Zhao et al., 2016; Sun et al., 2017). The data here indicated that AOS10 improved boar sperm quality. The data indicated that AOS could be used as a dietary additive for boars to improve their semen quality.

Gut microbiota play many physiological roles not limited to metabolic-related disorders such as obesity and diabetes (Bouter et al., 2017; Liu et al., 2017b), but also including nervous system and reproductive system-related diseases or conditions (Dai et al., 2015). Previously, we found that AOS benefited gut microbiota by the increase in the “beneficial” bacteria while the decrease in “harmful” bacteria in murine small intestines to rescue cell development (Zhang et al., 2020). Gut microbiota metabolize nutrients in the intestine and regulate intestinal metabolites to influence the blood metabolome (Tajima et al., 1999; Tusi et al., 2011). In turn, while traveling through body organs, blood metabolites can influence their development or induce disorders (Dai et al., 2015). It is known that metabolic regulation is essential for spermatogenesis (Cheng et al., 2010; Rato et al., 2010, 2012), and cholesterol and lipid homeostasis play a vital role in male fecundity (Cross, 1998; Ergün et al., 2007; Maqdasy et al., 2013; Lu et al., 2016; Kim et al., 2017). Sertoli cells act as nurse cells, providing the nutrients and energy for the process of spermatogenesis. Many other components such as hormones and endogenous or exogenous factors have a synergistic role in the homeostasis of metabolism in the testis and the progression of spermatogenesis (Rato et al., 2012). It has been shown that abnormal lipid metabolism in the reproductive system or blood contributes to human male infertility (Ergün et al., 2007; Lu et al., 2016; Kim et al., 2017). AOS could improve busulfan-damaged homeostasis of lipid metabolism in murine blood (Zhang et al., 2020). In the current investigation, the relative abundances of beneficial bacteria, such as *Lactobacillus,* were increased (1.35-fold), and the ratio of *Bacteroidetes*/*Firmicutes* was elevated by AOS. At the same time, AOS benefited boar sperm and blood metabolites by increasing levels of proline, lysine, retinol, betaine, etc. All the data indicated that AOS can boost boar semen quality through improving the gut microbiota, blood, and testicular metabolites.

The very interesting finding in the current study was that AOS benefited gut microbiota can continuingly improve boar semen quality after AOS treatment followed by 8 weeks of basal diet (without AOS supplementation). Because AOS cannot be directly absorbed into blood to reach the organs to affect their functions (Liu et al., 2019), the beneficial effects of AOS on sperm metabolites and semen quality may be due to the benefited gut microbiota. Otherwise, the effects cannot last for so long time. The data in the current investigation and our previous reports confirm that AOS benefit gut microbiota in mice and boars, and the benefited gut microbiota to increase semen quality.

## Conclusion

In summary, the beneficial impact of AOS on boar semen quality possibly through the positive changes in the gut microbiota and plasma/sperm metabolism was revealed. These improvements will large litter sizes and increase the economical supply of porcine meat for global consumption.

## Data availability statement

The datasets presented in this study can be found in online repositories. The names of the repository/repositories and accession number(s) can be found in the article/Supplementary material.

## Ethics statement

Ethical review and approval was not required for the animal study because we did not kill animals in the study, if necessary we will provide later. Written informed consent was obtained from the owners for the participation of their animals in this study.

## Author contributions

HH, YZo, BX, RZ, YJ, LC, HS, JT, BZ, and CG performed the experiments and analyzed the data. LC, HZ, and YZa designed and supervised the study and wrote the manuscript. All authors contributed to the article and approved the submitted version.

## Funding

This study was supported by the National Natural Science Foundation of China (32070536 and 31772408 to YZa; 31672428 to HZ).

## Conflict of interest

HS and JT were employed by YangXiang Joint Stock Company. BZ and CG were employed by Qingdao BZ Oligo Biotech Co., Ltd.

The remaining authors declare that the research was conducted in the absence of any commercial or financial relationships that could be construed as a potential conflict of interest.

## Publisher’s note

All claims expressed in this article are solely those of the authors and do not necessarily represent those of their affiliated organizations, or those of the publisher, the editors and the reviewers. Any product that may be evaluated in this article, or claim that may be made by its manufacturer, is not guaranteed or endorsed by the publisher.

## Footnotes

^1^ http://www.genome.jp/KEGG/pathway.html

## Supplementary material

SUPPLEMENTARY FIGURE S1 Sperm metabolite data. **(A)** PCA of sperm metabolites. **(B)** Quality control of sperm metabolite data. **(C)** Enriched pathways of changed sperm metabolites. **(D)** Correlation of sperm metabolite with each other.

SUPPLEMENTARY FIGURE S2 Blood metabolite data. **(A)** PCA of blood metabolites. **(B)** Quality control of blood metabolite data. **(C)** Correlation of blood metabolite with each other.

SUPPLEMENTARY FIGURE S3 Fecal microbiota data. **(A)** Quality control of fecal microbiota data. **(B)** The alpha index of the fecal microbiota. **(C)** PCA of blood metabolites. **(D)** LDA distribution and Cladogram. Linear discriminate analysis effect size (LEfSe) was performed to determine the difference in abundance; the threshold of LDA score was 4.0. **(E)** The ratio of *Bacteroidetes/Firmicutes*.
