# Supplementary material for: Alginate oligosaccharides increase boar semen quality by affecting gut microbiota and metabolites in blood and sperm
Source: Front Microbiol. 2022 Aug 22;13:982152. doi: 10.3389/fmicb.2022.982152 (PMC9441641; doi:10.3389/fmicb.2022.982152)
Supplement: SUPPLEMENTARY FIGURE S1 — Sperm metabolite data. (A) PCA of sperm metabolites. (B) Quality control of sperm metabolite data. (C) Enriched pathways of changed sperm metabolites. (D) Correlation of sperm metabolite with each other. [file Data_Sheet_1.ZIP › Table S1 primary antibody.docx]

**Table S1.** Primary antibody information

| **Gene symbol** | **Name** | **Cat. #** | **Predicted size** | **Source (Animal)** | **Company** |
| --- | --- | --- | --- | --- | --- |
| AKT1 | Protein kinase B | bs-0115R | 56kd | Rabbit (polyclonal) | Beijing Biosynthesis Biotechnology CO. |
| Actin | actin | Ab3280 | 42kDa | Rabbit (polyclonal) | Abcam |
| PKA | cAMP dependent protein kinase alpha catalytic subunit | bs-0520R | 40kd | Rabbit (polyclonal) | Beijing Biosynthesis Biotechnology CO. |
| P-ERK | phospho-Erk1 (Thr202 + Tyr204) | bs-1645R | 43kDa | Rabbit (polyclonal) | Beijing Biosynthesis Biotechnology CO. |
| ZAG | Zinc Alpha 2 Glycoprotein | bs-19382R | 32kDa | Rabbit | Beijing Biosynthesis Biotechnology CO. |
| CATSPER | CATSPER | bs-23326R | 90kDa | Rabbit | Beijing Biosynthesis Biotechnology CO. |
| Gelsolin | Gelsolin | bs-1160R | 80kDa | Rabbit | Beijing Biosynthesis Biotechnology CO. |
| ODF2 | Cenexin1 | bs-10309R | 91kDa | Rabbit | Beijing Biosynthesis Biotechnology CO. |
| p-PI3K | phosphorylated Phosphoinositide 3-kinase | bs-5571R | 80kd | Rabbit (polyclonal) | Beijing Biosynthesis Biotechnology CO. |
| p-AKT | phosphorylated AKT | bs-2720R | 56kd | Rabbit (polyclonal) | Beijing Biosynthesis Biotechnology CO. |
