# Supplementary material for: Alginate oligosaccharides increase boar semen quality by affecting gut microbiota and metabolites in blood and sperm
Source: Front Microbiol. 2022 Aug 22;13:982152. doi: 10.3389/fmicb.2022.982152 (PMC9441641; doi:10.3389/fmicb.2022.982152)
Supplement: SUPPLEMENTARY FIGURE S1 — Sperm metabolite data. (A) PCA of sperm metabolites. (B) Quality control of sperm metabolite data. (C) Enriched pathways of changed sperm metabolites. (D) Correlation of sperm metabolite with each other. [file Data_Sheet_1.ZIP › Table S2 Composition and nutrient analysis of basal diet.docx]

**Table S1** **Composition and nutrient analysis of basal diet**

| Ingredient | Content, % |
| --- | --- |
| Corn | 35.15 |
| Barley | 24.83 |
| wheat | 15.82 |
| Rice bran meal | 9.40 |
| Soybean meal | 7.90 |
| Soybean oil | 2.00 |
| L-lysine | 0.40 |
| Methionine | 0.14 |
| Threonine | 0.24 |
| Ground limestone | 1.44 |
| Monocalcium phosphate | 1.21 |
| Sodium chloride | 0.48 |
| Premix* | 1.00 |
| total | 100 |
| Nutrient, % |  |
| Calculated NE, kcal/kg | 2.24 |
| Crude protein, % | 14.50 |
| Crude fat, % | 3.22 |
| Crude ash, % | 6.18 |
| Crude fiber, % | 4.15 |

*: Premix provided the following minerals per kilogram: 17 mg Cu, 160 mg Fe, 140 mg Zn, 50 mg Mn, 0.50 mg I, 0.50 mg Se, and 0.22 mg Cr.
