# Supplementary figures and images for: Alginate oligosaccharides increase boar semen quality by affecting gut microbiota and metabolites in blood and sperm
Source: Front Microbiol. 2022 Aug 22;13:982152. doi: 10.3389/fmicb.2022.982152 (PMC9441641; doi:10.3389/fmicb.2022.982152)

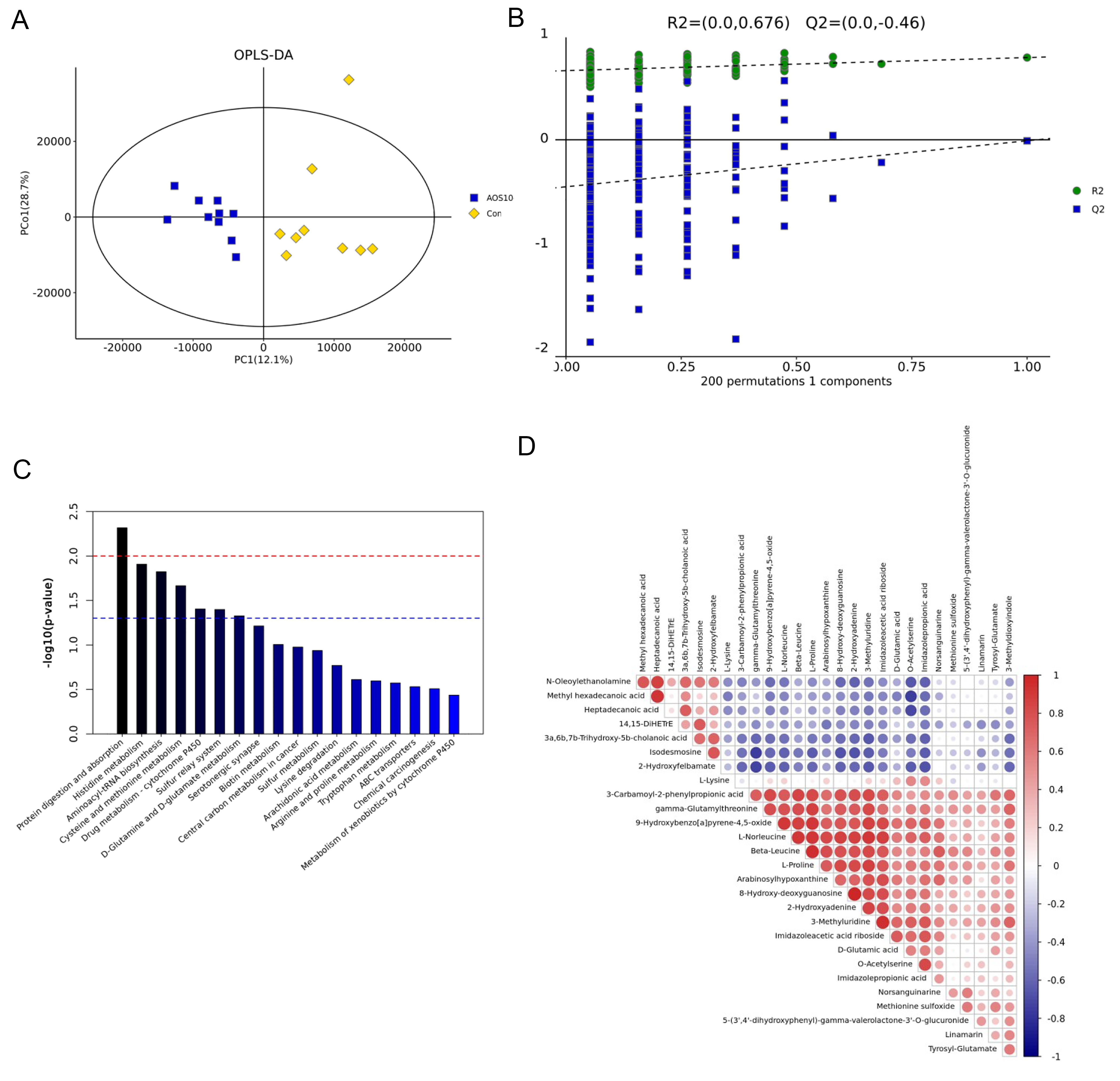

Supplement: SUPPLEMENTARY FIGURE S1 — Sperm metabolite data. (A) PCA of sperm metabolites. (B) Quality control of sperm metabolite data. (C) Enriched pathways of changed sperm metabolites. (D) Correlation of sperm metabolite with each other. [file Data_Sheet_1.ZIP › Fig. S1.tif]

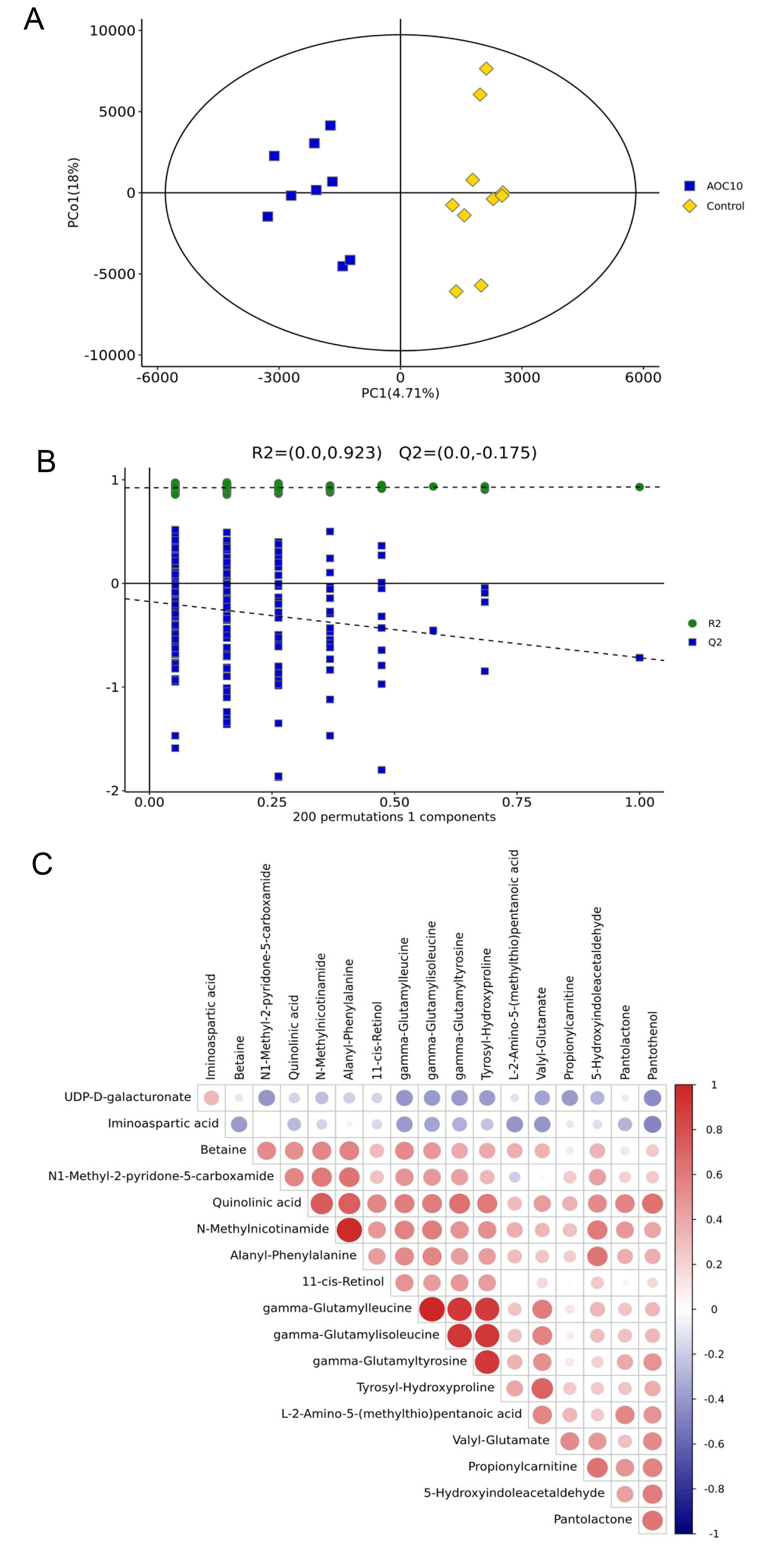

Supplement: SUPPLEMENTARY FIGURE S1 — Sperm metabolite data. (A) PCA of sperm metabolites. (B) Quality control of sperm metabolite data. (C) Enriched pathways of changed sperm metabolites. (D) Correlation of sperm metabolite with each other. [file Data_Sheet_1.ZIP › Fig. S2.tif]

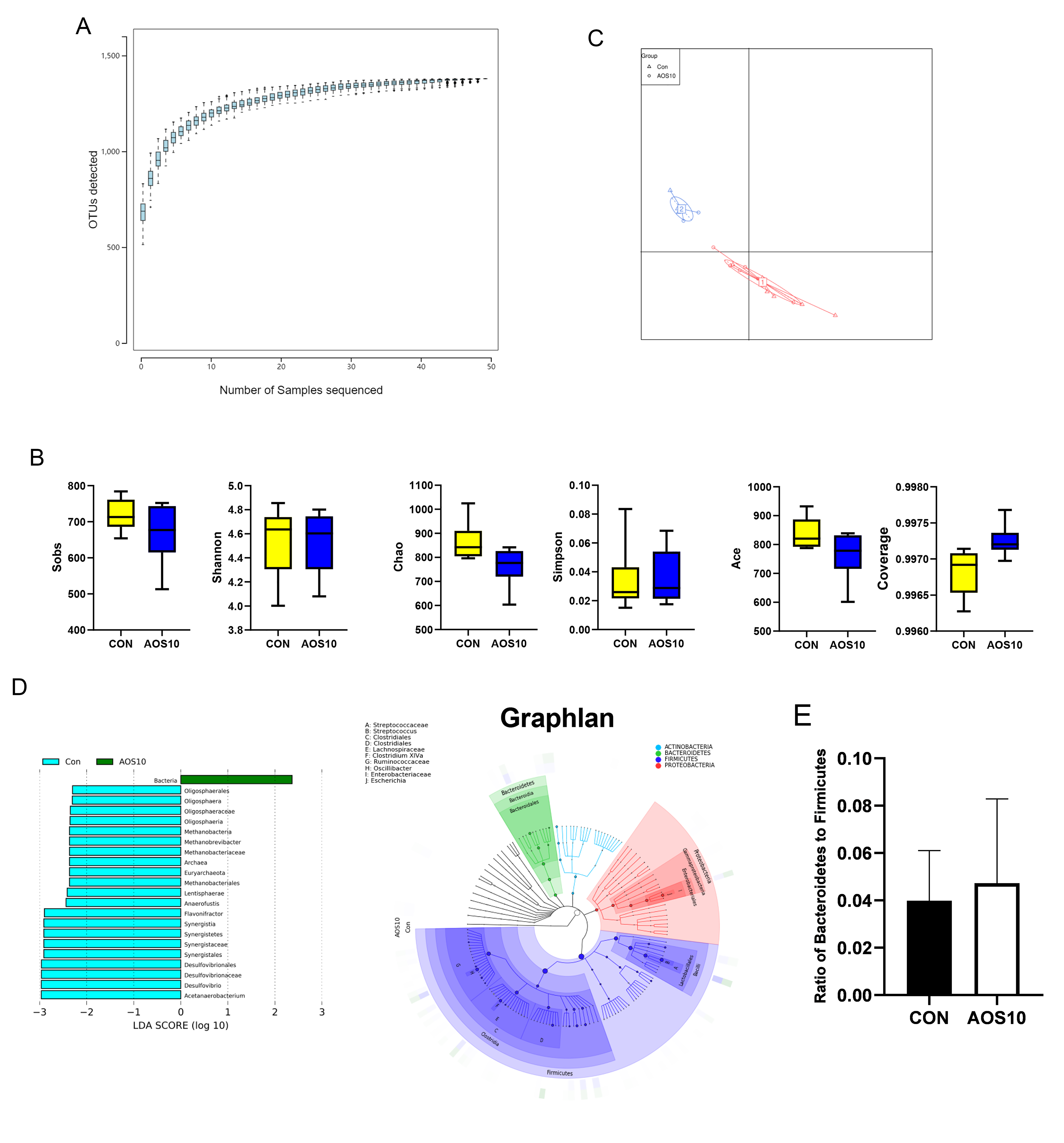

Supplement: SUPPLEMENTARY FIGURE S1 — Sperm metabolite data. (A) PCA of sperm metabolites. (B) Quality control of sperm metabolite data. (C) Enriched pathways of changed sperm metabolites. (D) Correlation of sperm metabolite with each other. [file Data_Sheet_1.ZIP › Fig. S3.tif]
